# Candidate Key Proteins in Thalamo-Amygdala Signaling in Tinnitus: A Bioinformatics Study

**DOI:** 10.3390/ijms27041854

**Published:** 2026-02-14

**Authors:** Johann Gross, Marlies Knipper, Birgit Mazurek

**Affiliations:** 1 Tinnitus Center, Charité-Universitätsmedizin Berlin, 10117 Berlin, Germany; birgit.mazurek@charite.de; 2 Leibniz Society of Science Berlin, 10117 Berlin, Germany; marlies.knipper@uni-tuebingen.de; 3 Department of Otolaryngology, Head and Neck Surgery, Tübingen Hearing Research Center (THRC), Molecular Physiology of Hearing, University of Tübingen, 72076 Tübingen, Germany

**Keywords:** amygdala, auditory perception, biomarker, synaptic activity, thalamus

## Abstract

With the aim of identifying key proteins that play a role in the disorder tinnitus, interactions between proteins involved in thalamo-amygdala signaling under conditions of normal hearing (NH), acoustic stimulation (AS), and tinnitus (Tin) were studied. Three gene lists compiled from the GeneCards database using keywords were characterized by analyses of overlap, protein–protein interaction (PPI) networks, and by protein-enrichment analysis. Key proteins were selected on the basis of the degree and combined score value of the corresponding PPI network. In the NH process, BDNF, CASP3, and PVALB were identified as high-degree proteins (HDPs). In the AS process, BDNF, PVALB, and DLG4 are the top three HDPs; in the Tin process, these are BDNF, APP, and TNF. In the Tin process, key proteins appear that differ pre- and postsynaptically from those detectable in NH or AS. The glucocorticoid receptor NR3C1 and its interaction with FKBP5, a glucocorticoid receptor-induced co-chaperone, appear to be of particular importance for the emotional aspects of tinnitus. In tinnitus, the HDPs, together with their high-score interaction proteins, indicate processes of chronic neurodegeneration and of changes in transcription, intercellular communication, and in the survival and growth of neurons.

## 1. Introduction

Tinnitus is the perception of sound (ringing, buzzing, hissing, etc.) in the ears or head without any external sound source; it is often a symptom of other issues, such as hearing loss or exposure to ototoxic medications, and is often associated with emotional dysfunction [1,2,3]. In about 1–5% of patients, tinnitus leads to emotional distress or cognitive dysfunction or interferes with daily life; this condition is termed tinnitus disorder. Around 10–20% of people with tinnitus experience a significant impact on their quality of life, often accompanied by anxiety, depression, or insomnia [4,5,6].

Several brain centers are involved in the acoustic signaling pathway from the cochlea to the cortex [7,8,9,10]. Sound waves are transformed into neural signals by hair cells in the Organ of Corti. These signals are carried via spiral ganglion neurons and their axons, forming the cochlear nerve, to the cochlear nuclei in the brainstem, the first central relay station. Signals are decoded for the sound frequency, level, and duration and transferred to the thalamus via the inferior colliculus (midbrain), a major integration center for auditory information.

The thalamus, specifically the medial geniculate body (MGB), is the main thalamic relay center for auditory information [11,12,13,14]. It refines and integrates signals before sending them to the cortex and other centers. Key functions of the thalamus in auditory perception are to transform sound for conscious perception. The MGB maintains dynamic communication loops with the auditory cortex in the form of the thalamocortical circuitry [15,16]. These loops support predictive coding and focus attention on important signals [17]. The MGB is highly plastic; changes in bottom-up signaling have been linked to tinnitus, where altered functional connectivity influences sound perception [18,19].

The amygdala is central to processing emotions like fear, anxiety, and stress, and it plays an important role in the emotional dimension of tinnitus. Tinnitus is being increasingly understood not just as an auditory issue but as a disorder involving the limbic system, which includes the amygdala [6,20,21]. The limbic system is involved in emotional responses, and its increased activity can amplify the perceived severity of tinnitus. Studies using fMRI have shown that compared to controls, people with chronic tinnitus may have reduced amygdala activation in response to emotionally evocative sounds [22,23]. This suggests a kind of neural adaptation, possibly to avoid constant emotional overload from the persistent phantom noise. Interestingly, the distress caused by tinnitus often correlates more strongly with limbic system activity than with the actual loudness of the sound [13,19,24].

While direct studies on this specific signaling route in tinnitus are still emerging, bioinformatics has already proven its worth in identifying key proteins and biological processes in related brain regions such as the thalamus and auditory system [25,26,27]. Databases like STRING (Search Tool for the Retrieval of Interacting Genes) and Cytoscape 3.9.1 help analyze and visualize how proteins interact via protein–protein interaction networks (PPI) in tinnitus-affected regions. This can highlight key proteins that may regulate synaptic transmission or neuroplasticity. Databases like DAVID (Database for Annotation, Visualization, and Integrated Discovery) and GeneCards (GC) allow researchers to identify biological processes via Gene Ontology (GO) enrichment analyses that are also relevant for understanding tinnitus. For diagnostics and therapy, it is important to understand the molecular processes in thalamo-amygdala signaling [28,29]. Bioinformatic analysis may illuminate which proteins contribute significantly to thalamo-amygdala signaling in tinnitus.

## 2. Results

### 2.1. Gene Lists Under Normal Hearing, Acoustic Stimulation, and Tinnitus

**Figure 1:**
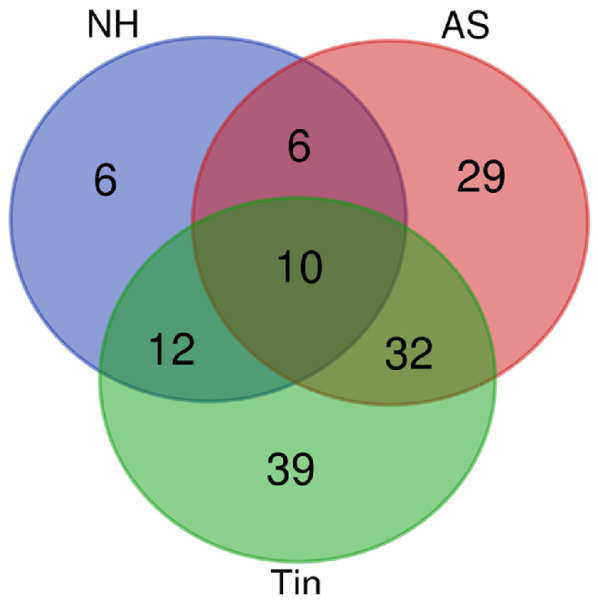
The Venn diagram of the genes involved in NH, AS and Tin processes. The overall number of unique elements is *n* = 134. Ten genes belong to all three processes: *BDNF-AS*, *BDNF*, *DLG4*, *SLC6A4*, *KCNH2*, *CACNA1A*, *IGF1*, *APOE*, *CASP3*, and *NR3C1*.

The number of genes involved in synaptic activity differs for normal hearing (NH, *n* = 34), acoustic stimulation (AS, *n* = 77), and tinnitus (Tin, *n* = 93), most probably limited by the current state of knowledge. The Venn diagram (Figure 1) indicates a remarkable overlap: 32 genes are present in the AS and Tin processes, indicating a clear overlap between them. The ten genes that belong to all three processes play an important role in the following aspects of synaptic activity: (a). *BDNF-AS*, *BDNF*, *DLG4* and *SLC6A4* are involved in synaptic transmission and plasticity. *BDNF-AS* is a long, non-coding RNA that suppresses BDNF transcription, with the consequence of potentially reducing synaptic transmission. Inhibition of *BDNF-AS* increases BDNF mRNA and activates neuronal development and differentiation. BDNF is a key protein in synaptic transmission and plasticity in the central nervous system, including in the auditory thalamus [30,31]. Both BDNF and *BDNF-AS* play important roles in the auditory pathway [32]. DLG4 (discs large MAGUK scaffold protein 4, PSD 95) is a postsynaptic scaffolding protein; it anchors NMDA and AMPA receptors and thus regulates excitatory synaptic strength and plasticity at glutamatergic synapses [33]. SLC6A4 is a serotonin transporter that influences serotonin levels in the synaptic cleft; it regulates serotonin reuptake and thus affects neurotransmission in the auditory thalamic circuits. The serotonin (5-HT) system seems to be a key regulator of emotional behavior in the amygdala [34]. It modulates circuits involved in mood, cognition, movement, arousal, and autonomic function [35]. (b). KCNH2 (ERG1; voltage-gated K^+^ channel) and CACNA1A (voltage-gated calcium channel) influence membrane excitability, firing patterns, and synaptic integration [36,37,38]. (c). IGF1, APOE, CASP3 and NR3C1 are expressed in normally functioning neurons and are important proteins in synaptic plasticity [39,40,41,42]. Specifically, IGF-1 (insulin-like growth factor 1) is a powerful regulator of synaptic activity, and a deficit in this protein has a profound impact on neurotransmission, mostly on excitatory synapses in both the developing and mature auditory system [39]. ApoE transports cholesterol and other lipids in the brain; it is mainly synthesized by astrocytes. ApoE is involved in several neuropathological changes such as neurogenesis and the modulation of synaptic plasticity [36,43]. CASP3 has been recognized as an “apoptotic” protein; however, in nervous tissue, it may be involved in important cellular processes not necessarily related to cell death. A concept on a new mechanism of synaptic plasticity modulation involving caspase-3 has been formulated, postulating a specific role of CASP3 in normal brain functioning [42]. NR3C1 is a glucocorticoid receptor responsible for stress-hormone signaling; it may affect auditory sensitivity and auditory learning. Altered NR3C1 expression has been observed in hearing disorders [44].

### 2.2. Networks and Key Proteins of Synaptic Activity Proteins

The structure of the three networks (NH, AS, and Tin;) is well reflected in the topological criteria (Table 1); they correspond to heterogenous (scale-free) networks of different sizes. Nodes and edges increase markedly from NH to AS to Tin, suggesting the progressive recruitment and integration of other proteins. Characteristic path length decreases in AS and Tin compared to NH, and the clustering coefficient rises from NH to Tin, suggesting increased local connectivity. The Tin network appears larger and more tightly clustered than the NH network.

**Figure 2:**
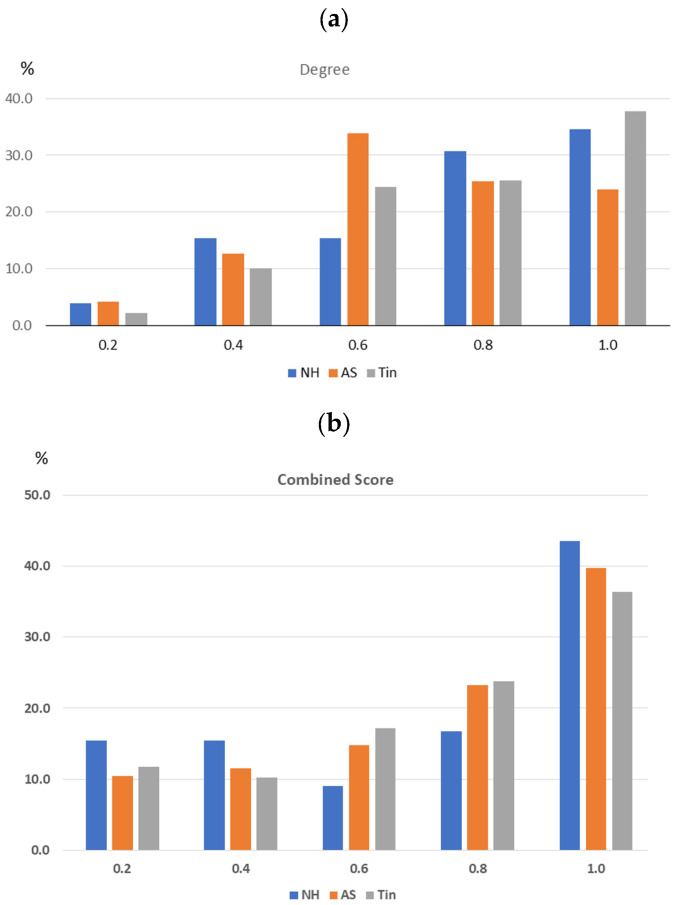
Frequency of the distribution of the degree (**a**) and CS (**b**) values of the NH, AS, and Tin networks. To calculate the frequency distribution of degree and CS values, five classes were created based on the 20th, 40th, 60th, 80th, and 100th percentiles. The 0.2 quantile class shows the highest degree and CS values.

Proteins with high-degree values (HDPs) and their high-score interaction proteins (HSIPs) are regarded as functionally important proteins in a PPI network, because they either connect many other proteins or indicate critical interaction paths. The combined score (CS) was used as an indicator for the HSIPs, because it integrates multiple types of evidence (experimental, curated, text mining, etc.) and is, for us, the most plausible criterion to obtain clinically important insights into the complex regulatory mechanisms characteristic of tinnitus. To select critical values for the HDPs and the HSIPs, we analyzed the frequency distribution of the degree and combined score (CS) values, creating five classes ranging from 0.2 to 1.0 (class 0.2 corresponds to the highest 20% of values of degree and CS) (Figure 2). Degree values increase steadily with class level, whereas the CS values are not as tightly coupled to the CS class. Correlation analysis indicates a moderate positive linear relationship between the two variables (Pearson correlation coefficient (r): 0.59, *p*-value = 0.02). The 0.2 quantile class of degrees corresponds to one (NH), three (AS) and two (Tin) proteins; the 0.2 quantile class of interactions corresponds to 12 (NH), 79 (AS), and 124 interactions. To limit the study, we selected the top three HDPs and their HSIPs with a CS value > 90th percentile as key proteins (Table 2). In previous work, we showed that the top two to three HDPs with their HSIPs can be used as key proteins indicating important biological functions [26].

In the NH process, BDNF, CASP3 and PVALB appear as the top three HDPs, respectively (Table 2). BDNF (brain-derived neurotrophic factor) is an important growth factor in the CNS. It regulates fundamental processes such as growth, differentiation, synaptic transmission, synaptic plasticity, and neuronal survival under normal and pathological conditions. In the auditory system, BDNF interacts closely with NTRK2 (neurotrophic receptor tyrosine kinase 2, TrkB) and GDNF (glial cell line-derived neurotrophic factor). The binding of BDNF to NTRK2 initiates intracellular signaling cascades in the MAPK, PI3K/Akt, and PLCγ pathways and strongly influences synaptic transmission and plasticity. GDNF supports synaptic maintenance, function, and plasticity. CASP3 (caspase 3) can play an important non-apoptotic role in synaptic transmission, and it is now recognized as a regulator of synaptic plasticity [42,43,45]. It interacts closely with MAPK8, a member of the MAP kinase family [46]. CASP3 can be active at synapses without killing the neuron, influencing transmission by modifying receptors and structural proteins. MAP kinases are involved in a wide variety of cellular processes, such as proliferation, differentiation, the regulation of transcription, and development. This kinase is activated by various cellular stimuli and targets specific transcription factors; it thus mediates immediate-early gene expression in response to cell stimuli. PVALB (Parvalbumin) is a calcium buffer in fast-spiking interneurons. It regulates intracellular calcium levels during neuronal firing and plays an important role in normal synaptic transmission [47].

In the AS process, BDNF, PVALB and DLG4 appear as the top three proteins, respectively. Similarly to the NH process, BDNF interacts closely with NTRK2 and, in addition, with MECP2 (methyl-CpG binding protein 2, epigenetic regulator), CREB1 (cAMP responsive element binding protein 1, transcription factor), and SLC6A4 (solute carrier family 6 member 4, serotonin transporter). PVALB interacts closely with CALB1, and both proteins are markers of inhibitory GABAergic neurons [38]. DLG4 (discs large MAGUK scaffold protein 4) interacts closely with SYP (Synaptophysin) and GRIN1 (glutamate ionotropic receptor NMDA type subunit 1), which is central to synaptic plasticity and long-term potentiation (LTP) [48]. NOS1 (nitric oxide synthase 1) produces NO as a retrograde messenger and is involved in synaptic plasticity and LTP [49]. SYNGAP1 is a GTPase-activating protein and is involved in the regulation of synaptic plasticity and neuronal excitability [50]. SHANK1 (SH3 and multiple ankyrin repeat domains 1) is a scaffolding protein, particularly at the postsynaptic density at glutamatergic synapses [51]. FMR1 (fragile X messenger ribonucleoprotein 1) is a multifunctional RNA-binding protein and is important for synaptic plasticity [52]. The serotonin receptor HTR2A (5-hydroxytryptamine receptor 2A) is involved in presynaptic modulation of chemical synaptic transmission, and GRM5 (glutamate metabotropic receptor 5) is a metabotropic glutamate receptor, whose signaling activates a phosphatidylinositol–calcium second messenger system; both play an important role in schizophrenia [53].

In the Tin process, BDNF, APP and TNF appear as the top three proteins, respectively. BDNF interacts closely with NTRK1, NTRK3, and, in addition, with CREB1, NTF3 (neurotrophin 3) and GDNF (glial cell line-derived neurotrophic factor). APP (amyloid precursor protein) regulates synapse formation and plasticity and is involved in Alzheimer’s disease [54,55]. APP interacts closely with ten proteins that participate in diverse biological pathways, including those implicated in neurodegenerative disease mechanisms [54]. TNF (tumor necrosis factor) interacts closely with JUN, FASLG, CASP3 and PTGS2. TNF is a multifunctional proinflammatory cytokine; it is released by glia and, in some cases, by neurons and can influence homeostatic plasticity by increasing the AMPA receptor content at excitatory synapses and decreasing the GABAA receptor content at inhibitory synapses [56]. FASLG (Fas ligand) is a TNF superfamily ligand that binds Fas (CD95); the primary function of this transmembrane protein is the induction of apoptosis triggered by binding to FAS [57]. FASLG activation of Fas receptors on neurons and glia can trigger apoptotic cascades via caspase-3. Low activation levels of CASP3 can modify cytoskeletal and scaffold proteins and can prune synapses and spines [58]. The prostaglandin synthase PTGS2 (COX-2) is upregulated in neuroinflammatory states and modulates neuroinflammation and cognitive dysfunction [59].

**Figure 3:**
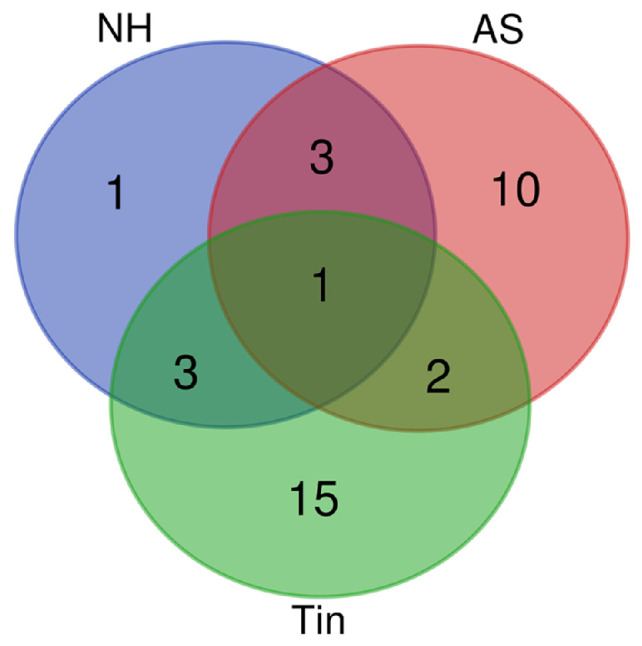
Venn diagram of the key proteins involved in normal hearing (NH), acoustic stimulation (AS), and tinnitus (Tin). The overall number of unique elements is *n* = 35.

In contrast to the multiple overlaps seen in the complete gene lists, the overlap of key proteins between NH, AS, and Tin in the Venn diagram is low, indicating that these proteins are specific to the respective processes. Only two proteins (SYP and CREB1) overlap between AS and Tin processes (Figure 3).

### 2.3. Genes Related to Cellular Components and Biological Processes in Normal Hearing, Acoustic Stimulation, and Tinnitus

**Figure 4:**
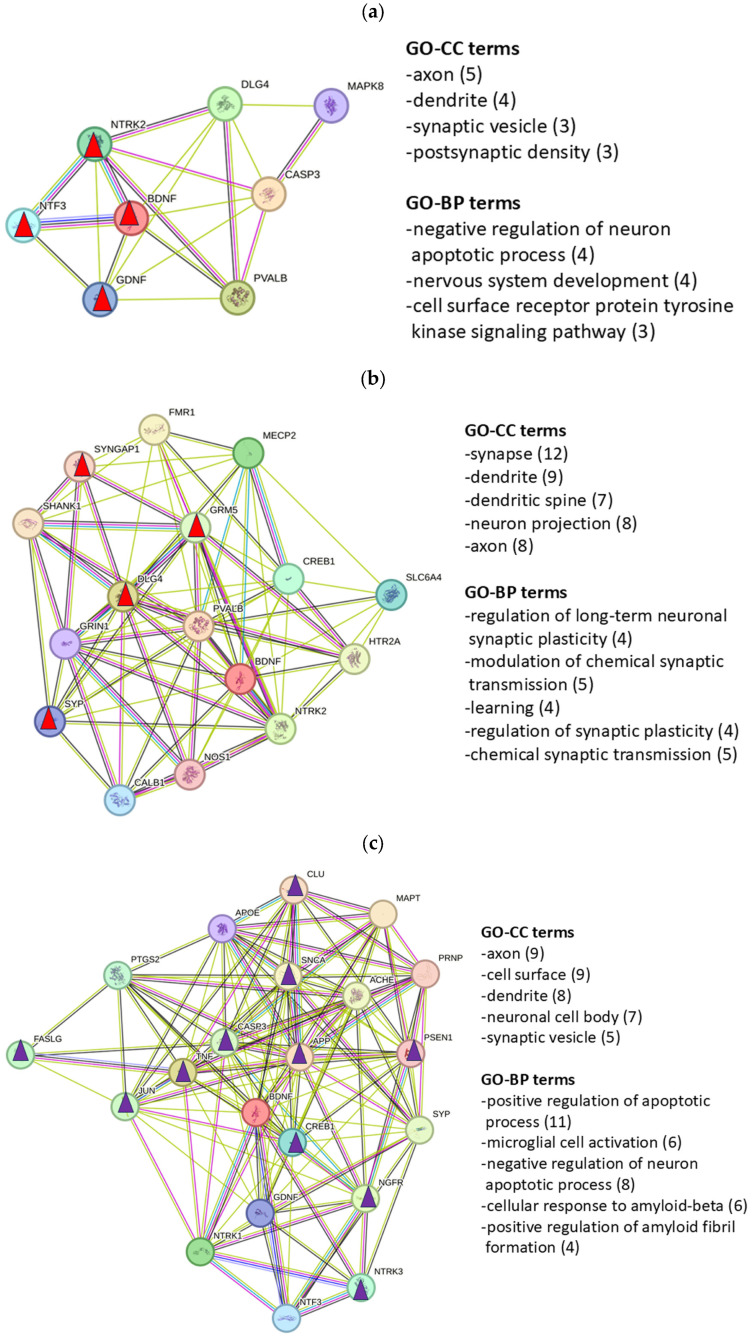
Networks and GO-BP terms reflected by the key proteins in the NH (**a**), AS (**b**), and Tin (**c**) processes. Triangles indicate HSIPs of the first-mentioned GO-BP terms (red—NH and AS; violet—Tin). In parentheses, the number of genes involved in the term are indicated. Total number of significant processes (Fisher’s test, *p* < 0.01): NH—8 proteins had 30 records; AS—16 proteins had 60 records; Tin—21 proteins had 166 records. Significance (Fisher’s test and Benjamini–Hochberg test): NH: GO-CC 2.6E − 6 to 2.5E − 3 and 1.4E − 4 to 3.2E − 2; GO-BP 1.2E − 5 to 6.0E − 4 and 1.6E − 3 to 3.7E − 2. AS: GO-CC 3.5E − 12 to 9.4E − 9 and 3.9E − 10 to 2.1E − 7; GO-BP 3.0E − 7 to 2.8E − 5 and 1.2E − 4 to 1.9E − 3. Tin: GO-CC 3.5E − 9 to 9.1E − 6 and 5.2E − 7 to 2.3E − 4; GO-BP 2.9E − 13 to 9.2E − 9 and 2.5E − 10 to 1.4E − 6.

To characterize the functional role of the genes and proteins present in the complete GC lists (Appendix A, Table A1**)** and the key protein lists (Table 2), we used the DAVID database for assignment of the enrichment of Gene Ontology terms for cellular components (CCs) and biological processes (BPs; Table 3 and Figure 4; Fisher’s test *p* < 0.01 and Benjamini–Hochberg *p*-value < 0.05). The cellular structures correspond in NH, AS, and Tin to GO-CC terms that characterize synaptic components involved in the hearing process. The GO-BP terms that characterize biological functions show clear differences in NH, AS, and Tin. The term “cellular response to amyloid-beta” in NH is unexpected; this GO term could reflect the diverse physiological functions of amyloid-beta, including its role in modulating synaptic transmission [60].

### 2.4. Networks of Key Proteins and Their Biological Processes in NH, AS, and Tin

To characterize the relevance of key proteins, the networks (NWs) of key proteins were constructed and the associated GO enrichment terms were determined (Figure 4a–c). The GO terms are partly identical to those of the complete networks (Table 3) but they partly differ in the sense of specifying the processes. The NH-NW (Figure 4a) corresponds to GO terms indicating protective and developmental processes important for normal functioning of the auditory system. The AS-NW (Figure 4b) comprises significantly more proteins than the NH-NW; the proteins of this NW correspond to GO terms such as “regulation of long-term neuronal synaptic plasticity” and “modulation of chemical synaptic transmission”, terms that are associated with adaptive processes to acoustic stimulation. The Tin-NW (Figure 4c) includes even more proteins than the AS-NW and focuses in particular on processes of apoptosis regulation and microglial cell activation.

### 2.5. NTRK2-, NTRK1- and NTRK3-Specific Networks Among NH, AS and Tin

**Figure 5:**
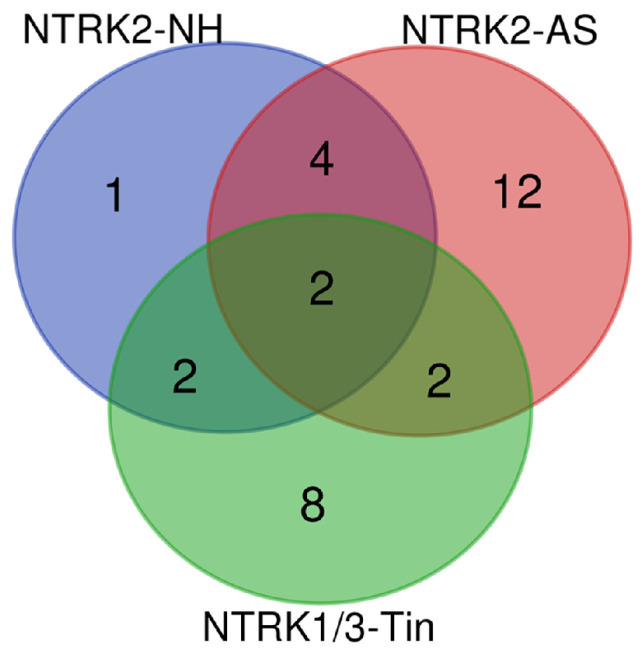
Venn diagram of the distribution of proteins interacting with NTRK2 and NTRK1/3. Total number of proteins with CS values > median (NH—560, AS—564, and Tin—581): 31.

It is remarkable that NTRK2 is an important key protein in NH and AS processes but not in the Tin process; instead, NTRK1 and NTRK3 (NTRK1/3) play the dominant neurotrophic role, probably indicating unchanged NTRK2 activity compared to NH. Thus, we analyzed important protein interactions (CS value > median) of NTRK2 in NH and AS and the interactions of NTRK1/3 in Tin. Two proteins, BDNF and IGF1, interact with NTRK1, NTRK2, and NTRK3 (Figure 5). The Venn diagram also shows that NTRK1/2/3 interact in NH, AS, and Tin with different proteins.

**Figure 6:**
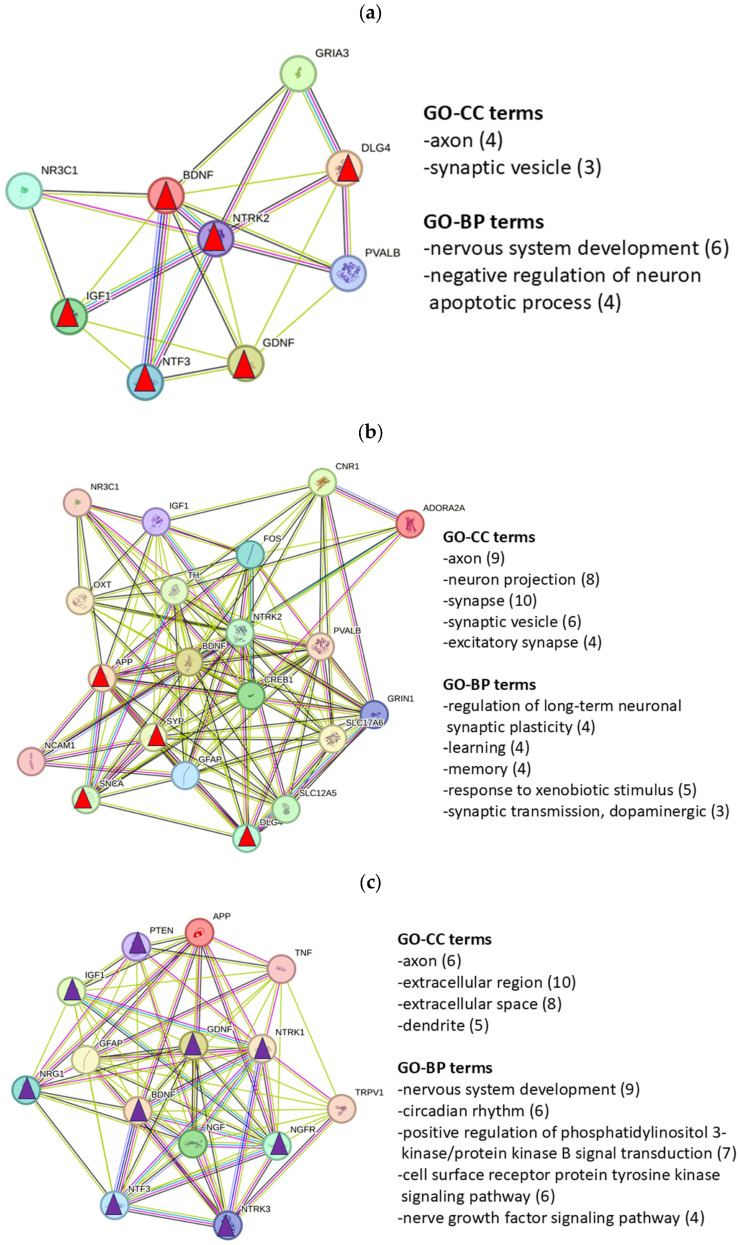
PPI networks of NTRK2 in the NH (**a**) and AS (**b**) processes and of NTRK1/NTRK3 in the Tin (**c**) process. Because of their overlap, the proteins of NTRK1 and NTRK3 are presented in one figure (**c**). Triangles indicate HSIPs of the first-mentioned GO-BP terms (red—NH and AS; violet—Tin). Significance (Fisher’s test and Benjamini–Hochberg test): NH: GO-CC 3.0E − 4 to 8.7E − 4 and 1.2E − 2 to 2.5E − 2; GO-BP 2.1E − 6 to 3.4E − 5 and 5.4E − 4 to 4.4E − 3. AS: GO-CC 1.9E − 9 to 2.7E − 6 and 1.7E − 7 to 7.4E – 5; GO-BP 5.3E − 7 to 8.4E − 5 and 3.1E − 4 to 1.8E – 3. Tin: GO-CC 4.3E − 7 to 2.2E − 3 and 3.5E − 5 to 3.6E − 2; GO-BP 1.3E − 8 to 5.1E − 7 and 7.6E − 6 to 6.1E − 5.

PPI networks of NTRK2 in the NH and AS processes and the network of NTRK1/3 in Tin were constructed to illustrate the interactions for characteristic GO terms. In the center of the NTRK2-associated NH and AS networks, BDNF and NTRK2 appear, and both show identical degree values (Figure 6a,b; NH–8 degrees; AS—19 degrees). In the center of the NTRK1/3 Tin network, BDNF, NGF, NTRK1 and GFAP appear, also with identical degree values (Figure 6c; 16 degrees).

The assignment of GO terms of the NTRK2-NWs and of the NTRK1/3-NW interacting proteins overlaps with the terms identified from the corresponding complete gene list and key protein list (Table 3 and Figure 4). Remarkably, the term “nervous system development” is associated with six genes in the NTRK2-NH network (BDNF, DLG4, GDNF, IGF1, NTRK2, and NTF3) and nine genes in the NTRK1/3-Tin network (instead of DLG4 and NTRK2, the genes NGFR, NRG1, NTRK1, NTRK3, and PTEN).

## 3. Discussion

Characterizing and quantifying protein–protein interactions within PPI networks associated with NH, AS, and Tin can provide valuable insights into the molecular mechanisms that influence tinnitus pathogenesis. Proteins (nodes) that have a particularly large number of connections (degrees) to other proteins and their high-score interaction proteins, together named key proteins, appear to play a special role in the molecular mechanism of biological processes. The analyses of appropriate protein lists using a combination of PPI networks and the enrichment analyses of the complete GC lists and key protein lists allows for predictions about key proteins that are involved in tinnitus.

### 3.1. PPI Networks and Their Topological Constants

The different numbers of proteins available for NH, AS, and Tin lead to networks of different sizes (Appendix A, Table A1). Comparing the topological constants of the NH, AS, and Tin networks reveals notable changes associated with tinnitus. The characteristic path length decreases in Tin compared to NH, implying the integration of new proteins and more efficient communication across the network through shorter average paths; this may reflect heightened synchrony or hyperactivity. The clustering coefficient increases in Tin relative to NH, indicating stronger local connectivity and suggesting the emergence of additional functional modules or feedback loops. This pattern suggests that Tin relies more on parallel processing rather than the hierarchical control observed in NH, where BDNF and NTRK2 emerge as the dominant single-node regulators.

### 3.2. GO Enrichment Analysis

GO enrichment analysis helps to identify biological processes, molecular functions, or cellular components in gene or protein lists. The results of the GO enrichment analyses of the complete GC gene list and the key protein list indicate similarities but also differences (Table 3 and Figure 4). The GO-CC terms of the NH, AS, and Tin complete protein lists indicate the important role of axons, synapses, and dendrites without characteristic differences between the three processes. In contrast, clear differences are observed in the GO-BP processes between NH, AS, and Tin. In NH (GC list), processes like the “cellular response to amyloid-beta” and “negative regulation of apoptotic processes,” appear, whereas in AS, the GO-BP terms “response to xenobiotic stimulus” and “locomotor behavior” appear, changes which could be induced by acoustic stimulation. In addition, the key protein list indicates more specific GO-BP terms such as “regulation of long-term neuronal synaptic plasticity” and “modulation of chemical synaptic transmission,” cellular activities induced by acoustic stimulation. The GO-BP terms associated with tinnitus show processes present in the NH (“cellular response to amyloid-beta”) and AS processes (“response to xenobiotic stimulus”) but also the GO-BP “positive regulation of apoptotic process” (in contrast to the term “negative regulation of apoptotic process” in NH). As a highly significant GO-BP process, the GO-BP term “microglial cell activation” appears in the key protein list and includes the proteins JUN (Jun proto-oncogene), AP-1 transcription factor subunit, APP (amyloid-beta precursor protein), CLU (clusterin), MAPT (microtubule-associated protein tau), SNCA (synuclein alpha) and TNF (tumor necrosis factor). Each of these proteins plays a distinct role in pathophysiological processes like neurodegeneration, inflammation, and cellular stress responses [61].

### 3.3. Role of NTRK1 and NTRK3 for Tin Networks

The present study suggests that in synaptic transmission under normal conditions, BDNF interacts primarily with NTRK2 and, under Tin conditions, primarily with NTRK1 and NTRK3. NTRK2 is the high-affinity receptor for BDNF, and under normal physiological conditions, this is the dominant signaling pathway. Under these conditions, BDNF has low or negligible affinity for NTRK1 (TrkA) and NTRK3 (TrkC) [62]. These receptors primarily bind other neurotrophins with high activity: NTRK1 shows high affinity for NGF (nerve growth factor) and NTRK3 shows high affinity for NT-3 (neurotrophin-3) [63,64]. The absence of NTRK2 and the presence of NTRK1 and NTRK3 in the Tin gene lists lead to the assumption of a special role for NTRK1 and NTRK3 in the Tin process (Figure 6). Overactivation of NTRK pathways has been implicated in neurological disorders such as epilepsy and in some neurodevelopmental conditions [65]. In the thalamus, this could manifest as abnormal rhythmic activity or sensory misprocessing. Both neurotrophins interact in tinnitus with NGF, NTF3, NRG1, and PTEN (Figure 6c). In the thalamo-amygdala signaling context, these interactions may influence the survival and connectivity of sensory neurons projecting from the thalamus to the amygdala, and NTF3 and NTRK3 may fine-tune synaptic strength and axonal targeting in this circuit [66,67,68,69]. In the NTRK1/NTRK3 network (Figure 6c), the proteins NGF, BDNF, NTRK1, NRG1, IGF1, NTF3, and GDNF show similar high-degree values. It may be that under these overlapping and dysregulated conditions of expression or activities of growth and neurotrophic factors, significant changes in synaptic activity and plasticity follow [5,70,71,72].

Of special interest is the appearance of APP (second highest HDP in Tin) and TRPV1 (transient receptor potential vanilloid 1) in the NTRK1/3 network. APP (amyloid precursor protein) is best known for its link to Alzheimer’s disease and is involved in neurite outgrowth, synaptogenesis, and synaptic stabilization [73]. Cleavage products of APP were used as neurotrauma biomarkers among military and law enforcement personnel exposed to occupational overpressure [74]. APP and its cleavage products may modulate synaptic transmission and plasticity. TRPV1 is a nonselective cation channel activated by capsaicin and endogenous lipids [75,76]. It can regulate neurotransmitter release at presynaptic terminals and affect postsynaptic excitability. TRPV1 is expressed in the amygdala and may modulate emotional responses by altering synaptic strength in thalamo-amygdala circuits.

### 3.4. Role of Key Proteins in Thalamo-Amygdala Signaling in NH, AS and Tin

**Figure 7:**
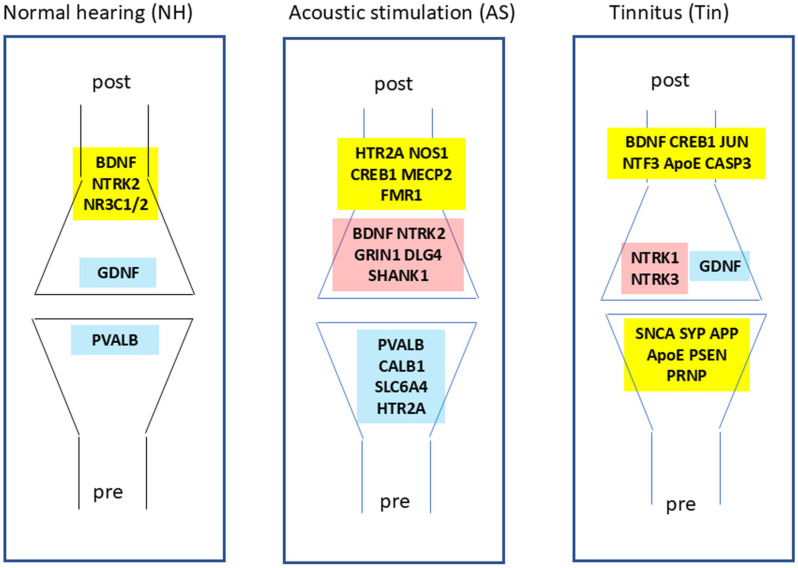
Distribution of key proteins in the pre- and postsynapse. Colors: blue—inhibitory; yellow—modulatory; red—excitatory.

Because key proteins appear to be indicators of important biological processes, we analyzed their distribution at the pre- and postsynaptic level of the synapse (Figure 7). In the NH process, PVALB (Parvalbumin) appears in the presynapse; it is a calcium-binding protein in fast-spiking GABAergic interneurons and regulates timing and inhibition in amygdala circuits [47]. PVALB interneurons gate thalamic sensory input to the amygdala, ensuring precise timing and preventing overexcitation. At the postsynaptic site, proteins appear that play an important role in the thalamo-amygdala pathway: BDNF/NTRK2 signaling enhances LTP in the lateral amygdala and is crucial for the learning of fear [77,78]. GDNF supports the survival of the GABAergic neurons responsible for inhibitory tone in the amygdala [79]; it may thus help maintain inhibitory balance, preventing hyperactivation of the amygdala in response to benign stimuli.

In the AS process, PVALB, CALB1, SLC6A4, and HTR2A appear in the presynapse. Similarly to the NH process, PVALB and CALB1 (Calbindin) act as calcium buffers that modulate excitability and protect neurons from calcium overload [47,80]. PVALB may ensure fast, inhibitory control over excitatory inputs, and CALB1 stabilizes calcium signaling for consistent neurotransmission. SLC6A4 (serotonin transporter) and HTR2A (5-hydroxytryptamine receptor 2A) mediate serotonin signaling, which enhances neural plasticity and affects sensitivity to environmental cues. SLC6A4 controls the serotonin reuptake at presynaptic terminals, clears serotonin from the synaptic cleft, and modulates serotonergic tone and presynaptic excitability in thalamic and amygdala circuits [81]. In the postsynapse, BDNF, NTRK2, GRIN1, DLG4, and SHANK1 appear as primarily excitatory key proteins [20,82,83]. Carrying out modulating functions within the thalamo-amygdala pathway are the key proteins NOS1, CREB1, MECP2, and FMR1 [84,85,86,87]. Together, these proteins can play an important role in the transmission and modulation of acoustic signals from the thalamus to the amygdala, influencing fear, anxiety, and emotion.

In the Tin process, key proteins that appear pre- and postsynaptically differ clearly from those detectable in NH or AS. At the presynaptic site, the proteins SNCA, SYP, APP, ApoE, PSEN and PRNP are active (Figure 7). SNCA (alpha-synuclein) and SYP (synaptophysin) are synaptic vesicle membrane proteins and are involved in the modulation of chemical synaptic transmission. APP (amyloid-beta precursor protein) is a cell-surface receptor and transmembrane precursor protein that is cleaved by secretases to form a number of peptides. ApoE (apolipoprotein) is associated with high-density lipoproteins (HDLs), and both APP and ApoE influence synapse formation and synapse organization. Both are integral components of the presynaptic membrane and contribute to the regulation of the synaptic vesicle cycle. PSEN (presenilin 1/2) is part of the γ-secretase complex that is involved in APP cleavage [55]. PRNP (prion protein) is involved in neurotransmission and plasticity [88,89]. The proteins APP, ApoE, and PSEN1 correspond in GO enrichment analysis to the biological processes “positive regulation of amyloid fibril formation” and “response to oxidative stress”. Together, these proteins indicate changes in the vesicle membrane proteins at the bases of neurodegenerative processes [90,91].

On the postsynaptic level, proteins appear that are involved in several regulatory networks. BDNF, NTRK1/3, and CREB1 proteins are involved in activity-dependent gene expression and synaptic strengthening; NTRK3 is an integral component of the postsynaptic membrane and is involved in the regulation of synaptic assembly. GDNF (glial cell line-derived neurotrophic factor) maintains inhibitory balance, preventing hyperactivation of the amygdala in response to excitatory stimuli. GDNF is also an important trophic factor essential for the survival of neurons [92]. The role of NTF3 is less clear. Following auditory deprivation of adult rats, changes in gene expression and interactions with specific gene promoters in the auditory cortex were observed [93]. The proteins BDNF, GDNF, MECP2, NTRK1, NTF3, and PSEN1 belong to the GO biological process “negative regulation of neuron apoptotic process”, and the proteins BDNF, NTRK1, NTRK3, and NTF3 belong to the term “cell-surface receptor protein tyrosine kinase signaling pathway”, indicating roles in neurogenesis and growth, transcription, and intercellular communication in tinnitus. Key proteins that are pre- and postsynaptically active indicate processes of chronic neurodegeneration in thalamo-amygdala signaling, which include the activation or the increase in the extent of amyloid fibril formation. Although the deposition of amyloid fibrils is a characteristic process in Alzheimer’s disease, amyloid formation is a normal process and it seems that the balance between amyloid formation and clearance in the thalamus is disrupted [73,74]. The aberrant signal transduction in the auditory centers in tinnitus and the imbalances in excitation and inhibition could induce excessive cell death, neurogenesis, and glia-supported neuroinflammation [91].

### 3.5. Role of Other Proteins in Thalamo-Amygdala Signaling

**Figure 8:**
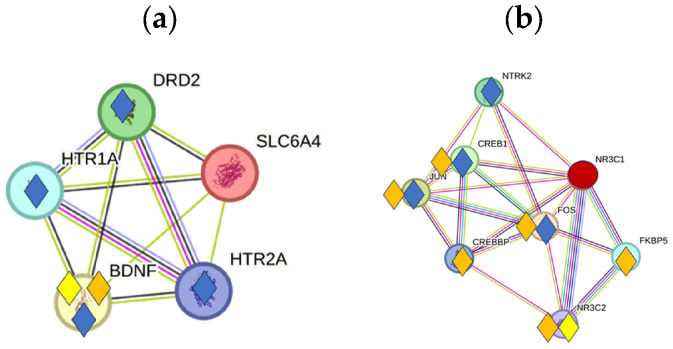
Interactions of SLC6A4 (**a**) and NR3C1 (**b**) with proteins in the NH, AS, and Tin processes. The squares indicate proteins with which SLC6A4 and NR3C1 interact with CS values > 90th percentile (NH—930, AS—896, and Tin—908). Colors: yellow—NH, blue—AS, and dark yellow—Tin.

The genes SLC6A4, HTR2A, and NR3C1 may play a special role in the emotional aspect of acoustic signals and tinnitus in the thalamo-amygdala circuitry. SLC6A4 (serotonin transporter) and NR3C1 (glucocorticoid receptor gene) are present in all three GC lists, both with a relatively high GC score, indicating a role in hearing under normal, stimulated, and tinnitus conditions. HTR2A only appears in the AS-GC list (Appendix A, Table A1). Possible relationships between SLC6A4 and the psychological symptoms of tinnitus are inferred from the influence of 5-HTTLPR polymorphism and selective serotonin reuptake inhibitors (SSRIs) on cognitive function and tinnitus distress [94,95]. The interactions of SLC6A4 and NR3C1 with other proteins in the NH, AS, and Tin processes are shown in Figure 8a. SLC6A4 shows close associations with BDNF under all conditions, with DRD2 and HTR1A or HTR2A2A only in the AS process.

The glucocorticoid receptor NR3C1 is a key player in the brain’s response to stress via the hypothalamic–pituitary–adrenal axis. NR3C1 at the postsynaptic level likely mediates stress hormone effects and acts as a potent regulator of transcription in the thalamus–amygdala circuit [96,97]. NR3C1 and NR3C2 integrate hormonal signals with neural activity, modulate synaptic plasticity and gene expression in neurons, and influence excitability and stress reactivity [96,98]. It is noteworthy that NR3C1 is detectable in all three GC lists (Appendix A, Table A1, Table A2 and Table A3) with a relatively high GC score but not in the key protein list. A more detailed analysis shows that the interactions of NR3C1 with the identified HDPs are below the selected critical value for HSIPs (>90th percentile). Because of its relevance for stress and emotion, we analyzed the interactions of NR3C1 in a separate subnetwork (Figure 8b). Using the 90th percentile as the critical CS value, NR3C1 interacts in NH with NR3C2, in AS with NTRK2 and different transcription factors (CREB1, JUN, CREBBP, and FOS), and in the Tin process with these previously mentioned transcription factors, NR3C2 and the protein FKBP5 (FKBP prolyl isomerase 5). FKBP5 is a glucocorticoid receptor-induced co-chaperone that reduces glucocorticoid receptor sensitivity and shapes stress reactivity [99,100]. NR3C1-FKBP5 interactions are only present in the Tin list (Table A3) and could be of importance in the stress perception in tinnitus [101,102].

To the best of our knowledge, none of these genes or proteins (NR3C1, SLC6A4, and HTR2A) are currently confirmed as tinnitus-susceptibility genes by GWAS or case reports. However, these proteins are clearly involved in some pathophysiological processes (stress response, synaptic signaling, and neurotransmission pathways) of tinnitus. NR3C1 modulates stress hormone signaling. Recently, it was shown that in a tinnitus group experiencing panic attacks, consistently higher mean methylation values of NR3C1 were observed across all CpGs compared to the tinnitus-only and control groups [103]. SLC6A4 and HTR2A regulate serotonergic signaling in auditory pathways (dorsal cochlear nucleus and inferior colliculus), but no association between the psychoacoustic parameters of tinnitus and SLC6A4 polymorphism has been observed [104]. Clifford et al. [105] identified 39 tinnitus loci, among them SLC6A4 but also BDNF, GDNF and FOS, identified here as key proteins.

The molecular mechanisms of stress perception in tinnitus are complex and include other receptors and, in addition to the thalamus and amygdala, other brain regions (e.g., cortex and hippocampus) [106,107,108]. In the present analysis, the distribution of glutamate receptors differs between NH, AS, and Tin processes (Appendix A, Table A1). In NH, GRIA3 appears; it is a subunit of AMPA-type glutamate receptors that are crucial for fast excitatory neurotransmission in the brain. Due to its expression in both the thalamus and amygdala, its role in thalamo-amygdala signaling is particularly relevant. In the AS gene list, GRIN1 appears as a key protein, together with GRM5 and GRM1. The interaction between GRIN1 and GRM1/GRM5 facilitates the induction of LTP and long-term depression (LTD), key mechanisms underlying learning and memory [109,110]. In the Tin process, six glutamate receptors are present in the corresponding list: four glutamate receptors also present in the NH and AS processes (GRIA3, GRIN1, GRM1, and GRM5) and two glutamate receptors that were not observed in the NH and AS processes (GRIA2 and GRIN2A). None of these receptors appear in the Tin process as key proteins.

The results are in agreement both with tinnitus theories that point to the role of neurodegeneration in tinnitus [13,111,112,113,114] and with theories that stress thalamocortical dysrhythmia, the interaction of separable subnetworks, and the topographic organization of neurons in the acoustic thalamus that project to the amygdala. [13,18,115,116,117,118].

### 3.6. Limitations

Bioinformatics is a powerful field for studying molecular mechanisms. Its principal limitation is the quality and actuality of the data: our results reflect the present state of scientific knowledge. We used databases that are well known and have been checked by the scientific community. By using a specific score, the GeneCards database selects genes that are specifically involved in biological symptoms, processes, and diseases. The present study is based on gene lists for NH, AS, and Tin, which were selected by keywords. Gene lists comprise genes that show relationships to the chosen keywords, in the sense of genetic relations, differential gene expression, and inferred knowledge from the scientific literature. The combination of keywords determines the number of genes that are involved in the process of interest; too large networks are unspecific, and too small networks are not representative. Key proteins, as used in this study, reflect dominant cellular components and biological processes in the tissues indicated, not in specific auditory centers or individual neurons. The fact that the GO terms between the complete list and the key protein list are in good agreement and that the results are in agreement with the literature knowledge supports the present approach. However, the results of a bioinformatic study cannot replace experimental data. They can give valuable hints for experimental studies, but these need to be confirmed experimentally, and our labs are not able to carry out these studies. In a previous study, we found that synaptic transmission in the thalamus is region-specific for NH, AS, and Tin for signaling to the auditory system and to the motor system in the cortex [26].

## 4. Material and Methods

In the present study, the presence of PPI networks, of key proteins, and of GO enrichment terms were analyzed under conditions of normal hearing (NH), acoustic stimulation (AS), and tinnitus (Tin), with the aim of better understanding the molecular processes of thalamo-amygdala signaling. Three gene lists were compiled from the GeneCards database (GC; https://www.genecards.org/) for the following keywords: (a). thalamus, “synaptic activity”, amygdala, and “normal hearing” (NH, 34 results); (b). thalamus, “synaptic activity”, amygdala, and “acoustic stimulation” (AS, 77 results); (c). thalamus, “synaptic activity”, amygdala, and tinnitus (Tin, 93 results). For the term “medial geniculate nucleus”, not enough data were available in the GC database (downloaded on 8 January 2026). These gene lists were characterized by analyses of gene overlap using Venn diagrams (http://bioinformatics.psb.ugent.be/webtools/Venn/, accessed on 10 February 2026) and by the construction of protein–protein interaction networks using the STRING database (Search Tool for the Retrieval of Interacting Genes; https://string-db.org/) [119]. For protein-enrichment analysis, we used the Database for Annotation, Visualization, and Integrated Discovery (DAVID; https://davidbioinformatics.nih.gov/tools.jsp, accessed on 10 February 2026) [120]. Fisher’s exact test and the Benjamini–Hochberg value were used for the significance of the GO terms (*p* < 0.01 for the gene lists and *p* < 0.05 for the keyword lists). The top five significant GO terms are indicated in the tables and figures; the total numbers of significant terms are indicated in the legends. The Cytoscape data analyzer (https://cytoscape.org/) was used to identify key proteins in the PPI network [121]. Key proteins were identified based on two criteria: node degree and edge combined score (CS). We hypothesized and found that the HDPs and the corresponding high-score interaction proteins (HSIPs), together named key proteins, play a functionally important role in the regulation of protein–protein networks [122,123]. Because of different biases within the list of proteins, and to limit the study, only the top three high-degree proteins (HDPs) were selected for analysis. We used the CS value of a protein pair as a criterion to identify HSIPs, with CS values > 90th percentile. The following databases were used for the brief definition and characterization of proteins and genes: https://www.ncbi.nlm.nih.gov/ (accessed on 13 December 2025); https://www.uniprot.org/uniprotkb/ (accessed on 27 November 2025); https://syngoportal.org (accessed on 13 December 2025); https://thebiogrid.org/ (accessed on 27 November 2025)

## 5. Conclusions

Key proteins that appear pre- and postsynaptically in the Tin process differ clearly from those detectable in NH or AS processes. On the presynaptic side, the proteins SNCA, SYP, APP, APOE, and PSEN are active. The proteins SNCA and SYP indicate changes in the vesicle membranes and activities. The proteins APP, APOE, PSEN1 intersect with tinnitus-related neurodegenerative processes, which include the activation of amyloid fibril formation. Postsynaptically active key proteins indicate changes in synaptic transmission by promoting long-term potentiation via increased NTRK1/NTRK3 activity, in combination with the activation of transcription and growth factors. In the thalamus, this might lead to increased synaptic density or the reorganization of circuits, potentially altering sensory processing. The interactions of the glucocorticoid receptor NR3C1 with transcription factors and FKBP5 appear as important proteins in thalamo-amygdala signaling that are responsible for the emotional aspects of tinnitus. The high interconnectivity between NTRK1/NTRK3, growth, and transcription factors may induce perturbations in the PPI network that are responsible for thalamo-amygdala signaling in tinnitus.

## Figures and Tables

**Table 1:** Topological criteria of the thalamo-amygdala networks.

Criteria	NH	AS	Tin
1. Number of nodes	26	71	90
2. Number of edges	78	751	1049
3. Mean number of neighbors	6.00	21.16	23.31
4. Characteristic path length	2.10 *	1.77 *	1.81 * ^#1^
5. Clustering coefficient	0.51 *	0.58 *	0.59 * ^#2^
6. Network heterogeneity	0.73	0.57	0.57
7. Network centralization	0.52	0.48	0.50

* *p* < 0.001 (degree-preserving permutation test); ^#1^ *p* < 0.02 vs. NH; ^#2^ *p* < 0.001 vs. NH (Cohen’s d test).

**Table 2:** Key proteins involved in thalamo-amygdala synaptic activity.

NH (90th Perc. CS > 930)		AS (90th Perc. CS > 896)		Tin (90th Perc. CS > 908)	
HDP (Degree)	HSIP (CS)	HDP (Degree)	HSIP (CS)	HDP (Degree)	HSIP (CS)
BDNF (18)	NTRK2 (999)	BDNF (54)	NTRK2 (999)	BDNF (67)	NGFR (999)
	NTF3 (991)		MECP2 (965)		NTRK1 (999)
	GDNF (963)		CREB1 (956)		NTRK3 (999)
			SLC6A4 (922)		CREB1 (956)
					NTF3 (991)
					GDNF (963)
CASP3 (14)	MAPK8 (943)	PVALB (45)	CALB1 (964)	APP (60)	ApoE (999)
					PSEN1 (999)
					PRNP (998)
PVALB (11)		DLG4 (42)	SYP (998)		CLU (998)
			GRIN1 (998)		MAPT (995)
	-		NOS1 (996)		SNCA (993)
	-		SYNGAP1 (996)		NGFR (959)
			SHANK1 (992)		ACHE (937)
			FMR1 (938)		SYP (918)
			HTR2A (915)		CASP3 (908)
			GRM5 (902)	TNF (52)	JUN (989)
					FASLG (984)
					CASP3 (939)
					PTGS2 (916)

In parentheses: degree values for HDPs and combined score values for HSIPs.

**Table 3:** GO terms for cellular components and biological processes of normal hearing, acoustic stimulation, and tinnitus.

Cellular Components	Biological Processes
Normal hearing (2.7E − 8 to 7.6E − 5; 3.8E- to 2.1E − 3) *	Normal hearing (3.7E − 5 to 1.7E − 4; 1.3E − 2 to 2.2E − 2)
-axon (9)	-cellular response to amyloid-beta (4)
-synapse (11)	-positive regulation of gene expression (7)
-dendrite (8)	-negative regulation of neuron apoptotic process (5)
-postsynaptic density (6)	-nervous system development (7)
-neuronal cell body (6)	-regulation of gene expression (6)
Acoustic stimulation (9.0E − 28 to 2.9E − 16; 2.5E − 25 to 1.4E − 14)	Acoustic stimulation (7.6E − 16 to 4.7E − 12; 1.2E − 12 to 1.4E − 9)
-synapse (36)	-response to xenobiotic stimulus (17)
-neuron projection (24)	-locomotory behavior (13)
-dendrite (25)	-chemical synaptic transmission (15)
-axon (20)	-memory (11)
-neuronal cell body (19)	-learning or memory (10)
Tinnitus (7.8E − 25 to 5.0E − 18; 2.5E − 22 to 3.3E − 16)	Tinnitus (4.5E − 18 to 2.3E − 13; 8.8E − 15 to 9.0E − 11)
-neuron projection (27)	-response to xenobiotic stimulus (20)
-axon (26)	-cellular response to amyloid-beta (12)
-dendrite (27)	-learning or memory (13)
-synapse (34)	-positive regulation of apoptotic process (18)
-neuronal cell body (22)	-chemical synaptic transmission (16)

* The GO terms are ordered according to their significance level (Fisher’s exact test *p*-value; Benjamini–Hochberg value); the number of genes involved in the term are given in parentheses. Total number of significant processes (Fisher’s test, *p* < 0.01): NH—26 proteins had 62 significant records, AS—71 proteins had 302 records, and Tin—89 proteins had 457 records.

## Data Availability

The original contributions presented in this study are included in the article. Further inquiries can be directed to the corresponding author.

## Appendix A

**Table A1:** Gene list for the normal hearing process in thalamo-amygdala signaling (NH, *n* = 34).

Gene	Name	Score
*SLC6A4*	Solute Carrier Family 6 Member 4	25.44
*BDNF-AS*	BDNF Antisense RNA	20.15
*BDNF*	Brain-Derived Neurotrophic Factor	13.66
*CERNA3*	Competing Endogenous LncRNA 3 For MiR-645	8.76
*DLG4*	Discs Large MAGUK Scaffold Protein 4	8.3
*APOE*	Apolipoprotein E	8.2
*PVALB*	Parvalbumin	7.62
*CHAT*	Choline O-Acetyltransferase	7.53
*CACNA1A*	Calcium Voltage-Gated Channel Subunit Alpha1 A	6.49
*NR3C1*	Nuclear Receptor Subfamily 3 Group C Member 1	5.79
*NR3C2*	Nuclear Receptor Subfamily 3 Group C Member 2	5.71
*NTRK2*	Neurotrophic Receptor Tyrosine Kinase 2	5.4
*UCN*	Urocortin	5.08
*GJA1*	Gap Junction Protein Alpha 1	4.79
*SNORD15A*	Small Nucleolar RNA, C/D Box 15A	4.01
*TRA-TGC7-1*	TRNA-Ala (Anticodon TGC) 7-1	3.76
*GPHN*	Gephyrin	3.44
*CAMK2G*	Calcium/Calmodulin Dependent Protein Kinase II Gamma	3.36
*GDNF*	Glial Cell Derived Neurotrophic Factor	3.13
*DNMT1*	DNA Methyltransferase 1	3.09
*NTF3*	Neurotrophin 3	3.09
*CASP3*	Caspase 3	3.09
*AQP4*	Aquaporin 4	2.59
*SOD2-OT1*	SOD2 Overlapping Transcript 1	2.38
*IGF1*	Insulin Like Growth Factor 1	2.13
*GRIA3*	Glutamate Ionotropic Receptor AMPA Type Subunit 3	2.09
*SMN1*	Survival Of Motor Neuron 1, Telomeric	2.01
*ATP6V1C1*	ATPase H+ Transporting V1 Subunit C1	1.87
*CYLD*	CYLD Lysine 63 Deubiquitinase	1.34
*MAPK8*	Mitogen-Activated Protein Kinase 8	1.28
*SLC8A1*	Solute Carrier Family 8 Member A1	1.04
*KCNH2*	Potassium Voltage-Gated Channel Subfamily H Member 2	0.86
*SLC12A2*	Solute Carrier Family 12 Member 2	0.82
*KCNJ6*	Potassium Inwardly Rectifying Channel Subfamily J Member 6	0.79

**Table A2:** Gene list for the acoustic stimulation process in thalamo-amygdala signaling (AS, *n* = 77).

Gene	Name	Score
*SLC6A4*	Solute Carrier Family 6 Member 4	28.09
*BDNF-AS*	BDNF Antisense RNA	21.43
*MAPT*	Microtubule Associated Protein Tau	15.56
*SNCA*	Synuclein Alpha	15.38
*LOC110806262*	Solute Carrier Family 6 Member 4 Gene Promoter	15.04
*BDNF*	Brain-Derived Neurotrophic Factor	14.66
*PSEN1*	Presenilin 1	13.14
*APP*	Amyloid-Beta Precursor Protein	11.82
*CRH*	Corticotropin-Releasing Hormone	11.11
*CALB1*	Calbindin 1	10.8
*FOS*	Fos Proto-Oncogene, AP-1 Transcription Factor Subunit	10.62
*DRD2*	Dopamine Receptor D2	10.4
*PVALB*	Parvalbumin	9.76
*HTR1A*	5-Hydroxytryptamine Receptor 1A	9.36
*SLC6A3*	Solute Carrier Family 6 Member 3	9.31
*HTR3A*	5-Hydroxytryptamine Receptor 3A	9.22
*GRIN1*	Glutamate Ionotropic Receptor NMDA Type Subunit 1	8.87
*APOE*	Apolipoprotein E	8.76
*HTR2A*	5-Hydroxytryptamine Receptor 2A	8.56
*DLG4*	Discs Large MAGUK Scaffold Protein 4	8.43
*CERNA3*	Competing Endogenous LncRNA 3 For MiR-645	8.22
*GFAP*	Glial Fibrillary Acidic Protein	7.66
*SLC17A6*	Solute Carrier Family 17 Member 6	7.6
*OPRM1*	Opioid Receptor Mu 1	7.34
*MECP2*	Methyl-CpG Binding Protein 2	7.3
*GRM5*	Glutamate Metabotropic Receptor 5	7.11
*GABBR1*	Gamma-Aminobutyric Acid Type B Receptor Subunit 1	7.09
*CREB1*	CAMP Responsive Element Binding Protein 1	6.86
*TH*	Tyrosine Hydroxylase	6.84
*NR3C1*	Nuclear Receptor Subfamily 3 Group C Member 1	6.55
*GABRA1*	Gamma-Aminobutyric Acid Type A Receptor Subunit Alpha1	6.52
*ACHE*	Acetylcholinesterase (Yt Blood Group)	6.24
*CRHR1*	Corticotropin-Releasing Hormone Receptor 1	6.13
*CACNA1A*	Calcium Voltage-Gated Channel Subunit Alpha1 A	5.86
*MAPK1*	Mitogen-Activated Protein Kinase 1	5.85
*GRM1*	Glutamate Metabotropic Receptor 1	5.75
*CNR1*	Cannabinoid Receptor 1	5.72
*RELN*	Reelin	5.66
*SYP*	Synaptophysin	5.53
*SRR*	Serine Racemase	5.52
*FMR1*	Fragile X Messenger Ribonucleoprotein 1	5.51
*EGR1*	Early Growth Response 1	5.44
*OXT*	Oxytocin/Neurophysin I Prepropeptide	5.19
*PPP1R1B*	Protein Phosphatase 1 Regulatory Inhibitor Subunit 1B	5.03
*NTRK2*	Neurotrophic Receptor Tyrosine Kinase 2	4.99
*DISC1*	DISC1 Scaffold Protein	4.8
*AVPR1A*	Arginine Vasopressin Receptor 1A	4.73
*ASIC1*	Acid-Sensing Ion Channel Subunit 1	4.54
*JUN*	Jun Proto-Oncogene, AP-1 Transcription Factor Subunit	4.45
*NRG1*	Neuregulin 1	4.3
*NCAM1*	Neural Cell Adhesion Molecule 1	4.13
*UBE3A*	Ubiquitin Protein Ligase E3A	3.99
*SCN1A*	Sodium Voltage-Gated Channel Alpha Subunit 1	3.84
*DRD1*	Dopamine Receptor D1	3.78
*TIRAP*	TIR Domain-Containing Adaptor Protein	3.76
*MAP2*	Microtubule-Associated Protein 2	3.56
*CHRNA7*	Cholinergic Receptor Nicotinic Alpha 7 Subunit	3.49
*CASP3*	Caspase 3	3.49
*NOS1*	Nitric Oxide Synthase 1	3.38
*PTEN*	Phosphatase And Tensin Homolog	3.38
*SOD1*	Superoxide Dismutase 1	3.22
*AQP4*	Aquaporin 4	2.99
*SYNGAP1*	Synaptic Ras GTPase-Activating Protein 1	2.97
*NCAN*	Neurocan	2.86
*SOD2-OT1*	SOD2 Overlapping Transcript 1	2.78
*ABCC1*	ATP Binding Cassette Subfamily C Member 1	2.71
*SLC12A5*	Solute Carrier Family 12 Member 5	2.71
*SHANK1*	SH3 And Multiple Ankyrin Repeat Domains 1	2.65
*ABCB1*	ATP Binding Cassette Subfamily B Member 1	2.24
*IGF1*	Insulin-Like Growth Factor 1	2.23
*ADORA2A*	Adenosine A2a Receptor	2.08
*ANKK1*	Ankyrin Repeat And Kinase Domain Containing 1	1.81
*KCNH2*	Potassium Voltage-Gated Channel Subfamily H Member 2	1.69
*NOS2*	Nitric Oxide Synthase 2	1.57
*CHRM2*	Cholinergic Receptor Muscarinic 2	1.55
*GSR*	Glutathione Disulfide Reductase	1.36
*KCNJ6*	Potassium Inwardly Rectifying Channel Subfamily J Member 6	1.27

**Table A3:** Gene list for the tinnitus process in thalamo-amygdala signaling (Tin, *n* = 93).

Gene	Name	Score
*SLC6A4*	Solute Carrier Family 6 Member 4	28.6
*BDNF-AS*	BDNF Antisense RNA	24.37
*BDNF*	Brain-Derived Neurotrophic Factor	18.2
*SNCA*	Synuclein Alpha	16.03
*LOC110806262*	Solute Carrier Family 6 Member 4 Gene Promoter	15.29
*MAPT*	Microtubule-Associated Protein Tau	15.15
*PSEN1*	Presenilin 1	12.38
*CACNA1A*	Calcium Voltage-Gated Channel Subunit Alpha1 A	11.81
*APP*	Amyloid-Beta Precursor Protein	10.91
*CALB1*	Calbindin 1	9.1
*NPY*	Neuropeptide Y	9.01
*PRNP*	Prion Protein (Kanno Blood Group)	8.86
*GFAP*	Glial Fibrillary Acidic Protein	8.71
*FOS*	Fos Proto-Oncogene, AP-1 Transcription Factor Subunit	8.51
*DLG4*	Discs Large MAGUK Scaffold Protein 4	8.39
*SLC1A3*	Solute Carrier Family 1 Member 3	8.36
*APOE*	Apolipoprotein E	8.34
*GRIN1*	Glutamate Ionotropic Receptor NMDA Type Subunit 1	8.03
*GDNF*	Glial Cell-Derived Neurotrophic Factor	7.87
*GABBR1*	Gamma-Aminobutyric Acid Type B Receptor Subunit 1	7.79
*GRIN2A*	Glutamate Ionotropic Receptor NMDA Type Subunit 2A	7.46
*MAPK1*	Mitogen-Activated Protein Kinase 1	7.35
*SLC17A6*	Solute Carrier Family 17 Member 6	7.2
*TH*	Tyrosine Hydroxylase	6.98
*CREB1*	CAMP Responsive Element Binding Protein 1	6.82
*GRM5*	Glutamate Metabotropic Receptor 5	6.52
*GRM1*	Glutamate Metabotropic Receptor 1	6.51
*GRIA2*	Glutamate Ionotropic Receptor AMPA Type Subunit 2	6.5
*SLC1A2*	Solute Carrier Family 1 Member 2	6.45
*SYP*	Synaptophysin	6.34
*NR3C1*	Nuclear Receptor Subfamily 3 Group C Member 1	6.23
*FKBP5*	FKBP Prolyl Isomerase 5	6.22
*EGR1*	Early Growth Response 1	6.17
*NGF*	Nerve Growth Factor	6.15
*NR3C2*	Nuclear Receptor Subfamily 3 Group C Member 2	6
*SCN1A*	Sodium Voltage-Gated Channel Alpha Subunit 1	5.97
*PTEN*	Phosphatase And Tensin Homolog	5.91
*ACHE*	Acetylcholinesterase (Yt Blood Group)	5.82
*TNF*	Tumor Necrosis Factor	5.81
*RELN*	Reelin	5.49
*MTOR*	Mechanistic Target Of Rapamycin Kinase	5.21
*NCAM1*	Neural Cell Adhesion Molecule 1	4.95
*IGF1*	Insulin-Like Growth Factor 1	4.9
*GPHN*	Gephyrin	4.77
*GJA1*	Gap Junction Protein Alpha 1	4.62
*SOD1*	Superoxide Dismutase 1	4.51
*GDF15*	Growth Differentiation Factor 15	4.34
*SLC12A2*	Solute Carrier Family 12 Member 2	4.24
*JUN*	Jun Proto-Oncogene, AP-1 Transcription Factor Subunit	4.16
*FMR1*	Fragile X Messenger Ribonucleoprotein 1	4.07
*NTF3*	Neurotrophin 3	4.02
*SNORD15A*	Small Nucleolar RNA, C/D Box 15A	3.9
*SLC1A1*	Solute Carrier Family 1 Member 1	3.89
*ABCC1*	ATP Binding Cassette Subfamily C Member 1 (ABCC1 Blood Group)	3.88
*CREBBP*	CREB Binding Lysine Acetyltransferase	3.81
*CASP3*	Caspase 3	3.74
*NRG1*	Neuregulin 1	3.61
*CAMK2G*	Calcium/Calmodulin-Dependent Protein Kinase II Gamma	3.45
*MAP2*	Microtubule-Associated Protein 2	3.41
*CASP9*	Caspase 9	3.38
*CLU*	Clusterin	3.37
*UCN*	Urocortin	3.35
*CYP46A1*	Cytochrome P450 Family 46 Subfamily A Member 1	2.82
*SNAP25*	Synaptosome-Associated Protein 25	2.81
*GSK3B*	Glycogen Synthase Kinase 3 Beta	2.77
*NTRK1*	Neurotrophic Receptor Tyrosine Kinase 1	2.69
*SIRT1*	Sirtuin 1	2.59
*SLC12A5*	Solute Carrier Family 12 Member 5	2.56
*CXCR4*	C-X-C Motif Chemokine Receptor 4	2.55
*GBA1*	Glucosylceramidase Beta 1	2.47
*CALCA*	Calcitonin-Related Polypeptide Alpha	2.41
*ABCB1*	ATP Binding Cassette Subfamily B Member 1	2.39
*PLAT*	Plasminogen Activator, Tissue Type	2.38
*VEGFA*	Vascular Endothelial Growth Factor A	2.34
*RAD51*	RAD51 Recombinase	2.2
*NGFR*	Nerve Growth Factor Receptor	2.19
*DNMT1*	DNA Methyltransferase 1	2.16
*SMN1*	Survival Of Motor Neuron 1, Telomeric	2.1
*SYNGAP1*	Synaptic Ras GTPase-Activating Protein 1	2.06
*DNM3*	Dynamin 3	2.03
*PTGS2*	Prostaglandin-Endoperoxide Synthase 2	1.97
*ITPR1*	Inositol 1,4,5-Trisphosphate Receptor Type 1	1.75
*MAPK8*	Mitogen-Activated Protein Kinase 8	1.53
*TRPV1*	Transient Receptor Potential Cation Channel Subfamily V Member 1	1.51
*DNM1*	Dynamin 1	1.49
*KCNH2*	Potassium Voltage-Gated Channel Subfamily H Member 2	1.38
*UCHL1*	Ubiquitin C-Terminal Hydrolase L1	1.32
*FASLG*	Fas Ligand	1.2
*BIN1*	Bridging Integrator 1	1.05
*NFE2L2*	NFE2-Like BZIP Transcription Factor 2	1.05
*NTRK3*	Neurotrophic Receptor Tyrosine Kinase 3	1.03
*ABCA2*	ATP Binding Cassette Subfamily A Member 2	0.86
*AMPH*	Amphiphysin	0.85
